# Supplementary material for: WordCommentsAnalyzer: A windows software tool for qualitative research
Source: F1000Res. 2018 Sep 4;7:536. Originally published 2018 May 3. [Version 2] doi: 10.12688/f1000research.14819.2 (PMC6137411; doi:10.12688/f1000research.14819.2)
Supplement: Supplementary file 3 [file f1000research-7-17614-s0002.tgz › b2a79496-4ada-47c4-9682-a99ce867836d.zip!/Eight Iranian Health Journals/Yafteh _33.docx]

© Yafteh  . This work is licensed under CC BY 4.0 (https://creativecommons.org/licenses/by/4.0/).

Volume & Issue: دوره 12 - شماره 3

Title: بررسی چاقی واختلال تحمل گلوکز در خانواده درجه اول زنان مبتلا به سندرم تخمدان پلی کیستیک مراجعه کننده به درمانگاههای زنان وابسته به دانشگاه علوم پزشکی شیراز،در سال1387

http://yafte.lums.ac.ir/article-1-318-fa.html

بیماری تخمدان پلی کیستیک یکی از شایع ترین اختلالات غدد داخلی در بین بیماران قبل از یائسگی است که شیوع بین المللی آن 4 تا 12 درصد است. ترکیبی از عدم تخمک گذاری مزمن، تخمدان پلی کیستیک، نازایی، اختلال قاعدگی، هیرسوتیسم، هایپرآندروژنیسم و چاقی ممکن است از ویژگی های آن باشد. این بیماری جنبه ژنتیکی دارد و در مطالعات مختلف اختلالات مشابهی در فامیل درجه اول آنها دیده شده است . مواد و روش ها: مطالعه یک پژوهش مورد-شاهدی است که در سال 87 بر روی 107 نفر از وابستگان درجه یک افراد مبتلا به این سندرم و 107 نفر به عنوان گروه کنترل باروش نمونه گیری تصادفی ساده مبتنی بر هدف انجام گرفته است. پس از شناسایی مبتلایان سندرم تخمدان پلی کیستیک وابستگان درجه اول آنها ( پدر،مادر،خواهر و برادران) مورد مصاحبه قرارگرفته ، شاخص توده بدنی و شاخص دور کمر به باسن افراد در هر دو نمونه خون جهت بررسی سطح سرمی تحمل گلوکز گرفته شد. بحث و نتیجه گیری: وابستگان درجه یک زنان مبتلا به سندرم تخمدان پلی کیستیک در معرض خطر ابتلاء به افزایش چاقی و چاقی مرکزی و اختلال تحمل گلوکز می باشند یافته ها: گروه آزمایش از نظر ابتلا به چاقی (30≤BMI) و چاقی مرکزی ، میزان اختلال تحمل به گلوکز (IGT) و دیابت با توجه به معیارهای سازمان بهداشت جهانی در گروه ازمایش بالاتر از افراد مشابه در گروه کنترل بود و این تفاوت از نظر آماری معنی دارنبود . میانگین قند خون ناشتا(FBS) درپدر،مادر، برادر و خواهران گروه آزمایش به طور معنی داری بالاتر بود (001/0=p) .با توجه به ازمون کای اسکور ارتباط معنی داری بین چاقی ( 2kg/m 30BMI≥) ، چاقی مرکزی با عدم تحمل به گلوکز ودیابت تیپ2 در دو گروه آزمایش وکنترل وجود داشت.

Title: کلونینگ و توالی‌یابی ژن کامل راپتری دو (ROP2) از توکسوپلاسماگوندی

http://yafte.lums.ac.ir/article-1-319-fa.html

توکسو پلاسما گوندی یک تک یاخته داخل سلولی می‌باشد که باعث بیماری توکسو پلاسموزیس در انسان و حیوانات می‌شود. بیمارانی که نقص سیستم ایمنی دارند، عفونت مزمن این بیماری می‌تواند دوباره فعال شده و باعث آنسفالیت شود که اغلب کشنده می‌باشد. پروتئین راپتری 2 توکسو پلاسما گوندی(ROP2) یکی از میانجی‌های مهم در آمیزش ارگانل-PVM می‌باشد. پروتئین ROP2 در بدن انسان توسط کلون Tcell شناسایی می‌شود . همچنین ROP2 برای Bcell دارای اپی توپ می‌باشد. همه این ویژگی های ROP2 باعث شده که از این پروتئین به عنوان کاندید برای تهیه واکسن های نوترکیبی و کوکتلی علیه توکسوپلاسموزیس استفاده شود. بحث و نتیجه گیری: نتایج این تحقیق نشان داد که با موفقیت ژن کامل ROP2 در داخل پلاسمید pTZ57R/T کلون شده است و از این پلاسمید نوترکیب (pT-ROP2) می توان برای ساختن DNA واکسن بر علیه توکسوپلاسموزیس استفاده کرد. یافته ها: این پژوهش نشان داد که ژن کامل ROP2 در داخل پلاسمید pTZ57R/T کلون شده و پلاسمید نوترکیب (pT-ROP2)را تشکیل می‌دهد. توالی‌یابی ژن کامل ROP2 که در داخل وکتور pTZ57R/T کلون شده است، نشان دادکه توالی ژن کامل ROP2 از سوش بسیار بیماریزای توکسوپلاسما گوندی (معروف به سوش RH)شباهت زیاد 98 درصدی با سوش RH موجود در بانک ژنی دارد. (Gen bank Accession No.Z36906.1). مواد و روش ها: در این مقاله کلونینگ و توالی یابی ژن کد کننده پروتئین کامل ROP2 از توکسوپلاسما گوندی توصیف خواهد شد. در این تحقیق ، ابتدا DNA ژنومی توکسوپلاسما گوندی استخراج شده و از آن برای تکثیر ژن کامل ROP2 با استفاده از پرایمرهای اختصاصی طراحی‌شده استفاده گردید.سپس محصول PCR در داخل وکتور pTZ57R/T کلون شده و متعاقبا پلاسمید کلون شده در باکتری E.Coli ترانس‌فورم شده و به دنبال آن پلاسمید حاوی ژن کامل ROP2(pT-ROP2)که توسط هضم آنزیمی و PCR تایید شده بود، از باکتریهای ترانس‌فورم شده، استخراج گردید و از آن برای توالی‌یابی ژن کامل ROP2 استفاده گردید

Title: اثربخشی آموزش مهارت‌های اجتماعی بر کاهش اعتیادپذیری در دانش‌آموزان دختر مقطع پیش دانشگاهی شهر تهران

http://yafte.lums.ac.ir/article-1-320-fa.html

ترکیب سنی جمعیت کشور ، اولویت بخشی به راهبردهای پیشگیرانه اعتیاد به مواد مخدر را آشکار می سازد . هدف پژوهش ، بررسی اثربخشی آموزش مهارت‌های اجتماعی بر کاهش اعتیادپذیری دانش‌آموزان دختر درمقطع پیش‌دانشگاهی شهر تهران است. بحث و نتیجه گیری: برگزاری دوره های آموزش مهارت های اجتماعی برای مبارزه با گرایش نوجوانان به مصرف مواد مخدر باید مطمح نظر گروه های مرجع همچون مشاوران ، مربیان مدارس و برنامه ریزان اجتماعی قرار گیرد. بعلاوه این روش می تواند به منزله رهیافتی مقابله ای در آموزش نوجوانان مورداستفاه قرار گیرد. یافته ها: تحلیل داده ها بیانگر تفاوت معنی دار بین نتایج آزمون ها در دو گروه کنترل و آزمایشی بود و نشان داد که آموزش مهارت‌های اجتماعی باعث کاهش اعتیادپذیری ، آمادگی به اعتیاد و کاهش اعتراف به آن در نوجوانان می‌شود مواد و روش ها: برای جمع آوری اطلاعات تحقیق ، در مرحله پیش آزمون 300 دانش آموز دختر و از طریق روش نمونه گیری خوشه ای چند مرحله ای انتخاب گردیدند. سپس در مرحله پس آزمون در هریک از دو گروه کنترل و آزمایشی ، 15 دانش آموز شرکت و به منظور جمع آوری داده ها از آزمون t مستقل برای تعیین معناداری تفاوت میانگین‌ها استفاده گردید

Title: توزیع فراوانی نارسایی حاد کلیوی در بیماران بستری در بخش مراقبتهای ویژه مرکز پزشکی الزهرا (س) اصفهان

http://yafte.lums.ac.ir/article-1-321-fa.html

بحث و نتیجه گیری: درصد فراوانی نسبی ARF بیانگر فراوانی قابل توجه آن در بیماران بستری در ICU می باشد . با افزایش سن فراوانی ARF افزایش یافته است که از این نظر مشابه با مطالعات قبلی بوده و احتمالاً با شدت بیماری زمینه ای و کاهش فانکشن کلیوی در این گروه سنی ارتباط می یابد . گرفتاری مردان بیش از زنان بود و احتمالا ً بدلیل افزایش بروز تروما و جراحی در مردان است . شایع ترین علل پررنال ARF از دست رفتن خون و مایعات با حجم زیاد بوده است . میزان مرگ و میر در این مطالعه بیشتر از مطالعات قبلی بوده و از نظر آماری اختلاف معنی دار بوده است(034/0 p=) . شدت ضایعات و اختلالات همراه این بیماران در میزان مرگ و میر مؤثر است .با توجه به اینکه ARF یکی از عوارض شایع و مهم در ICU محسوب می شود دقت در بررسی های لازم جهت ثبات وضعیت همودینامیک, تجویز مایعات در حد کفایت و تعدیل دوزاژ دارو و... جهت تشخیص سریع و درمان به موقع اینگونه بیماران ضروری است . مواد و روش ها: در این مطالعه توصیفی ـ تحلیلی , پرونده 1383 بیمار بستری در ICU جراحی طی سال های 80 – 1375 مورد بررسی قرار گرفت . یافته های لازم بر اساس سن , جنس , علت بستری , اتیولوژی و مرگ و میر در فرم جمع آوری اطلاعات وارد گردید و مورد تجزیه و تحلیل آماری قرار گرفت . یافته ها: فراوانی نسبی نارسایی حاد کلیوی 9/25 درصد بود که شایعترین علت آنرا علل پررنال ( 9/57 درصد ) تشکیل می داد . از علل پررنال , درناژ جراحی , خونریزی , و تروما فراوان ترین علل بودند . فراوان ترین گروه سنی ، سن بالای 70 سال ( 6/25 درصد ) و کمترین فراوانی مربوط به سنین 40 ـ 31 سال (13/6 درصد) بود . میانگیـن سن کـل بیمـاران 9 5/ 36 و میانگیـن سنی بیمـاران مبتلا به ARF 8/5 52 سال بود (05/0 p <) . گرفتاری مردان ( 3/30 درصد) بیشتر از زنان ( 9/19 درصد) بود (05/0 p < ) .میزان مرگ و میر ARF 9/57 درصد در مقایسه با سایر بیماران ( 7/12 درصد) بود (05/0 p < ) .از آنجائیکه نارسایی حاد کلیوی (ARF) باعث افزایش مرگ و میر بیماران و همچنین افزایش زمان بستری در بیمارستان و بالا رفتن هزینه های درمانی آنها میگردد , اطلاع دقیق از توزیع فراوانی , میزان مرگ و میر , نسبت جنسی و سایر خصوصیات نا رسایی کلیوی در بخش مراقبت های ویژه در جهت پیشگیری , درمان و مراقبت بهتر از بیماران و نیز پیش بینی امکانات و نیروی آموزش دیده نقش مهم و غیر قابل انکاری می تواند ایفا کند و در این راستا انجام این بررسی و مطالعه لازم و منطقی به نظر می رسد .

Title: تاثیر برنامه آموزشی" کسب مهارتهای بالینی در اورژانس" بر یادگیری دانشجویان رشته پزشکی

http://yafte.lums.ac.ir/article-1-322-fa.html

توانمندسازی و کسب مهارت های لازم در دانشجویان پزشکی، بخش اساسی آموزش پزشکی را تشکیل می دهد. وجود هر گونه مشکل در آموزش بالینی، کارآیی و بازده این بخش از آموزش را کاهش می دهد. عدم توانائی دانشجویان پزشکی در این زمینه می تواند از ضعفها و مشکلات آموزشی در دانشگاههای کشور محسوب گردد. در این راستا دانشگاه های علوم پزشکی کشور اقدام به راه اندازی مراکز آموزش مهارتهای بالینی نموده اند. بحث و نتیجه گیری: با توجه به نتایج این بررسی می توان گفت گذراندن دوره های آموزشی تئوری توام باعملی در زمینه اورژانس های شایع بیمارستانی می تواند تاثیرات مثبت معنا داری را در ارتقا سطح علمی و تصمیم گیری و اقدام به موقع و مناسب دانشجویان پزشکی در هنگام مواجهه با بیماران نیازمند خدمات اورژانسی داشته باشد. یافته ها: این تحقیق نشان داد که در کلیه اقدامات بالینی در اورژانس، اعم از سوند گذاری، تزریقات، بخیه زنی، آْتل بندی و دیگر اقدامات ضروری اورژانس، دانشجویان پس از طی دوره آموزشی تئوری و عملی زیر نظر استاد مربوطه، در پس آزمون نمرات بسیار مطلوبی در مقایسه با پیش آزمون کسب نمودند. مواد و روش ها: این مطالعه به صورت مداخله ای از نوع پیش آزمون- پس آزمون بوده و بر روی همه دانشجویان مقطع فیزیوپاتولوژی و مقطع کارورزی که قبلا دوره فیزیوپاتولوژی را گذرانده بودند انجام شد. قبل از شروع دوره آموزشی و پس از طی دوره آموزش تئوری و عملی در زمینه مهارتهای اورژانس از قبیل سوند گذاری، آتل بندی، تزریقات و بخیه زدن، پرسشنامه ای دو بخشی شامل مشخصات فردی و همچنین سوالات مربوط به مهارتهای بالینی اورژانس توسط هر دانشجو تکمیل گردید. در پایان اطلاعات وارد نرم افزار کامپیوتری شده و آنالیز گردیدند. روائی محتوائی پرسشنامه توسط اساتید صاحبنظر تائید شده و پایائی آن پس از انجام مطالعه اولیه در یک نمونه تصادفی 20 نفره با محاسبه ضریب همبستگی آلفای کرونباخ مقدار 93/0 محاسبه گردید.

Title: تاثیر اسانس مرزه خوزستانی(Satureja Khuzestaniea) بر مهار تغییرات گلومرولی در رتهای دیابتی –نفرکتومی یکطرفه

http://yafte.lums.ac.ir/article-1-323-fa.html

نفروپاتی دیابتی علت شایع رسیدن به مرحله نهایی بیماری کلیوی است . اسانس مرزه خوزستانی برای اولین بار بعنوان آنتی اکسیدان ،آنتی دیابت و ضد التهاب در مهار نفروپاتی دیابتی بکار گرفته شد. بحث و نتیجه گیری: اسانس مرزه خوزستانی بطور معنی داری (05/0 >p) میتواند هیپرتروفی گلومرولی ،گسترش مزانژالی را مهار و حجم مویرگ گلومرولی را در رتهای دیابتی حفظ نماید. یافته ها: هیپرتروفی گلومرولی ، گسترش مزانژالی و کاهش مویرگهای گلومرولی در گروههای درمانی با اسانس مرزه خوزستانی بطور معنی داری در مقایسه با گروه دیابتی بدون درمان مهار شده بود ولی با سطح این متغیرها در گروه کنترل تفاوت معنی داری داشت. سطح سرمی مالون دی آ لدهید در گروههای تحت درمان بطور معنی داری در سطح گروه کنترل حفظ شده بود(05/0 > p). مواد و روش ها: چهل رت نر مورد جراحی نفرکتومی چپ قرار گرفتند.رتها بطور تصادفی به چهار گروه (هر گروه 10 راس) تقسیم شدند ، گروه یک کنترل، گروه دوم دیابتی بدون درمان ، گروههای سوم و چهارم گروههای درمانی با اسانس مرزه به ترتیب با دوز درمانی 250ppm و 500ppm در آب خوردن .در گروههای دوم،سوم و چهارم با تزریق زیر جلدی آلوکسان دیابت القا گردید.بعداز8 هفته درمان مالون دی الدهید سرمی اندازه گیری شد.برشهای پارافینی از کلیه تهیه و بطریقه پاس رنگ امیزی گردید.حجم گلومرول ، حجم مزانژال داخل گلومرولی و حجم مویرگی گلومرولی با روشهای استریولوژیک برآورد گردید.اطلاعات به کمک نرم افزار SPSS13 و تست ناپارا متری من-ویتنی در05/0 p< مورد مقایسه قرار گرفتند.

Title: بررسی تاثیر ماساژپشت بر فشار خون و نبض رادیال بیماران مبتلا به پرفشاری خون اولیه مراجعه کننده به کلینیک قلب درمانگاه تخصصی و فوق تخصصی بیمارستان شهدای عشایر شهر خرم آباد

http://yafte.lums.ac.ir/article-1-324-fa.html

پرفشاری ‌خون اختلالی است که در اثر تنش یا استرس ایجاد شود. در بسیاری ‌از ‌افراد راهبردهای رفتاری نظیر آرام‌سازی، ماساژ وغیره درکنترل ‌پاسخ افراد به ‌استرس و در نتیجه کاهش فشار‌خون موثر می باشد. با توجه به ‌اثرات بیشتر مداخله درمانی ترکیبی بر مبنای روشهای رفتاری نسبت به کاربرد دارو درمانی محض انتظار می روداستفاده از تکنیکهای مذکور از جمله ماساژ در کاهش فشار خو ن موثر واقع شود. مواد و روش ها: در این ‌مطالعه نیمه‌تجربی بر‌اساس‌نتایج‌مطالعه ی مقدماتی، ‌90 بیمار مبتلا به پرفشاری خون اولیه انتخاب شدند. فشار خون و نبض رادیال این بیماران طی20 جلسه و به مدت 5 هفته قبل و پس از ماساژ پشت در روزهای شنبه و سه شنبه و بدون مداخله در روزهای یکشنبه و چهارشنبه کنترل و ثبت گردید. به منظور ‌جمع‌آوری داده‌ ها ‌از‌ پرسشنامه ‌ای شامل اطلاعات فردی، چک لیست ثبت فشار‌خون و نبض‌رادیال و فشار سنج ثابت برای کلیه بیماران‌استفاده شدوآنالیز آماری با کمک نرم افزار stata انجام شد. یافته ها: نتایج مطالعه نشان داد فشار خون سیستول و دیاستول پس از ماساژ به ترتیب 3/6 و 9/3 میلی مترجیوه(001/=0p) و نبض رادیال نیز پس از ماساژ به مقدار 9/2 ضربه در دقیقه کاهش یافت(001/0= p). بحث و نتیجه گیری: نتایج به دست آمده،حاکی‌از‌موثر‌بودن‌ماساژ‌در کاهش فشار‌خون و نبض‌رادیال بیماران مبتلا به پرفشاری‌خون‌اولیه بود. ‌استفاده‌ از ‌روشهای‌کنترل‌استرس ‌همچون ماساژ‌یک شیوه‌ی ‌قابل ‌قبول‌، ساده ‌و ‌قابل‌آموزش به‌خانواده‌ها جهت کنترل هر‌چه بیشتر بیماری می‌باشد.

Title: ارتباط پوسیدگی دندان با نمایه توده بدنی و عادات غذایی در کودکان مراجعه کننده به کلینیک دندانپزشکی دانشگاه علوم پزشکی تبریز

http://yafte.lums.ac.ir/article-1-338-fa.html

با توجه به اهمیت عادات غذایی و نقش احتمالی چاقی در ایجاد پوسیدگی دندان، مطالعه حاضر با هدف بررسی ارتباط شاخص پوسیدگی دندان در کودکان با نمایه توده بدنی و عادات غذایی در کلینیک دندانپزشکی دانشگاه علوم پزشکی تبریز انجام شد. مواد و روش ها: در این مطالعه مقطعی- تحلیلی 202 کودک 12-3 ساله بطور تصادفی انتخاب شدند. به منظور بررسی پوسیدگی دندان از شاخص تعداد دندان‌های پوسیده، کشیده شده و پرشده و برای ارزیابی عادات غذایی از پرسشنامه بسامد خوراک نیمه کمی استفاده گردید. از آزمون همبستگی و آنالیز رگرسیون جهت تجزیه و تحلیل داده ها استفاده شد. بحث و نتیجه گیری یافته‌های مطالعه نشان داد که با افزایش مصرف میوه، میزان پوسیدگی دندان کاهش یافته و از میان مواد غذایی بررسی شده، مصرف آجیل پیشگویی‌کننده معنی‌دار تعداد دندان‌های پوسیده بود. یافته ها: میانگین شاخص تعداد دندان‌های پوسیده، کشیده شده و پرشده در کودکان مورد بررسی 80/3±61/7 بود. همبستگی معکوس معنی‌داری میان این شاخص و سن(176/0- = r) و مصرف میوه (155/0- = r) وجود داشت (05/0p<). همبستگی معنی‌داری میان پوسیدگی دندان با نمایه توده بدنی بدست نیامد. بر اساس مدل نهایی رگرسیون نیز متغیرهای سن، شغل مادر و بسامد مصرف آجیل (مغزها) پیشگویی‌کننده‌های معنی‌دار تعداد دندان‌های پوسیده بودند.

Title: بررسی کنترل و نظارت بر مواد غذایی عرضه شده به دانش آموزان و میزان آگاهی تغذیه ای بهداشتی اولیاء و مربیان مدارس محدوده پایگاه تحقیقات جمعیتی خرم آباد در سال تحصیلی 86-1387

http://yafte.lums.ac.ir/article-1-339-fa.html

تغذیه مناسب از مهم ترین نیازهای تامین سلامت جسمی، فکری و به عبارتی رکن اساسی سلامت جامعه است. ارائه رژیم غذایی مناسب به کودکان، نگهداری و توزیع مطلوب، همچنین کنترل محل نگهداری و توزیع مواد غذایی در مدارس بسیار مهم است. لذا پژوهش حاضر با هدف بررسی کنترل و نظارت بر نحوه عرضه و توزیع مواد غذایی به دانش آموزان و میزان آگاهی تغذیه ای بهداشتی اولیاء و مربیان مدارس ابتدایی خرم آباد انجام گرفت. وکارشناسان بهداشت اهمیت دارد. یافته ها: از مجموع 39 مدرسه 7/29 درصد دارای بوفه بودند از این تعداد تنها 5/40 درصد دارای مربی بهداشت بودند. تعداد دانش آموزان مدارس مزبور به طور میانگین 6/107±7/175 بوده که از 53 تا 454 نفر متغیر بوده است. سطح آگاهی اولیاء و مربیان مدارس نسبت به نحوه نظارت و کنترل بر عرضه مواد غذایی در بوفه ها 5/61 درصد نسبتا خوب بودند به طوری که بین دارا بودن بوفه و آگاهی اولیاء و مربیان مدارس نسبت به نحوه نظارت و کنترل بر عرضه و توزیع مواد غذایی ارتباط معنی داری وجود داشت (025/0 = P) . نظارت کارشناسان بهداشتی بر عرضه مواد غذایی در بوفه ها 5/14 درصد در حد ضعیف بوده است. بحث و نتیجه گیری: این بررسی نشان دادداشتن مکانی مناسب وبهداشتی برای عرضه وتوزیع مواد غذائی درمدارس ضروری است .باتوجه به نیاز دانش اّموزان به میان وعده غذائی مناسب در محیط مدرسه نظارت بیشتر مسئولین مدرسه مواد و روش ها: مطالعه حاضر با روش توصیفی- مقطعی روی 39 مدرسه ابتدایی با 5695 دانش آموز دختر و پسر پایه های اول تا پنجم دبستان های ناحیه یک شهرستان خرم آباد انجام شد .برای بررسی وضعیت نظارت بر عرضه و توزیع مواد غذایی به دانش آموزان و سنجش میزان آگاهی های تغذیه ای بهداشتی اولیاء و مربیان سرشماری به عمل آمد و نمونه ها ی والدین از طریق تلفیقی از نمونه گیری طبقه ای، خوشه ای دو مرحله ای و سرانجام سیستماتیک انتخاب شدند.ابزار جمع آوری اطلاعات از طریق دو پرسشنامه خودساخته بود که با مراجعه حضوری, مشاهده و مصاحبه تکمیل گردید . سپس جهت توصیف داده ها از جداول توزیع فراوانی و آزمون های استقلال مجذور کای دو و نیز از نرم افزار SPSS نسخه 15 استفاده شد.
